# *BRCA1/2* Mutation Status Impact on Autophagy and Immune Response: Unheralded Target

**DOI:** 10.1093/jncics/pkaa077

**Published:** 2020-08-24

**Authors:** Susan Morand, Laura Stanbery, Adam Walter, Rodney P Rocconi, John Nemunaitis

**Affiliations:** Department of Internal Medicine, University of Toledo, Toledo, OH, USA; Gradalis, Inc, Carrollton, TX, USA; Promedica Health System, Toledo, OH, USA; University of South Alabama - Mitchell Cancer Institute, Mobile, AL, USA; Gradalis, Inc, Carrollton, TX, USA

## Abstract

BRCA1 and possibly BRCA2 proteins may relate to the regulation of autophagy. Autophagy plays a key role in immune response from both a tumor and immune effector cell standpoint. In cells with *BRCA* mutations, increased autophagy leads to elevated expression of major histocompatibility complex class II but may cause subclonal neoantigen presentation, which may impair the immune response related to clonal neoantigen visibility. We review evidence of BRCA1/2 regulation of autophagy, immune response, and antigen presentation.

Autophagy refers to the process by which a cell consumes its own constituents (1). It is increasingly evident that basal levels of autophagy may play a more important role than previously thought regarding sensitivity and/or resistance to immunotherapy, maintaining homeostasis by recycling cytosolic material, regulating metabolism, and eliminating harmful free radicals (2). Autophagy also plays a key role as a tumor suppressor (3-6). In the absence of autophagy, proto(oncoproteins) accumulate, exerting effects on cell growth, progression through the cell cycle, or angiogenesis, among other hallmarks (7-12). Similarly, impaired autophagy allows defective organelles to accumulate. Particularly, the accumulation of mitochondria in the absence of mitophagy results in increased reactive oxygen species and further damages DNA (13-15). Several studies demonstrate increased autophagy in cancer stem cells, during the Warburg effect, in anoikis and metastasis, and in resistance to chemotherapy (16-19). However, autophagy has a key role in major histocompatibility complex (MHC) processing (20-22) and permits the mounting of intracellular material onto MHC class II (MHC-II), which is traditionally thought of as the bearer of extracellular threats (23). Furthermore, autophagy communicates closely with the exosomal and endosomal pathway, thereby modulating the intercellular exchange of material, including tumor antigens and a further relationship to MHC-I display along with other immunomodulatory molecule expression (1,24). Together, these actions have important consequences on antitumor immunity.

## Autophagy Pathway

Autophagy begins with 3 principal steps: initiation, expansion, and formation of the autophagosome (1). During initiation, a phagophore is derived from the endoplasmic reticulum. In expansion, the phagophore approaches cytosolic material and expands. Finally, the phagophore completely envelopes the cytosolic contents to form the autophagosome. To complete autophagy, the autophagosome may fuse with a lysosome, which subsequently degrades the material and makes it available for processing via MHC-II. Alternatively, the autophagosome may fuse with an endosome to form an amphisome, which fuses with the lysosome to complete the process (1). Otherwise, it may engage in exosome transfer, in which it secretes its material to the extracellular matrix (1,24-26). Depending on the derivate cell, these exosomes may be referred to as tumor exosomes (TEXs) or dendritic cell exosomes (1).

The machinery for autophagy and exosome production overlap considerably. In the total absence of one process, the other process is incapable of occurring (24,26). Similarly, when conditions including hypoxia, chemotherapy, and endoplasmic reticulum stress occur, both autophagy and exosomal production are increased in response (24). Homeostasis exists between the 2 pathways, which share endosomes as a substrate (24,26). With activation of autophagy, intravesicular bodies (IVBs) preferentially fuse with autophagosomes to undergo degradation by lysosomes, thereby decreasing flux down the exosomal pathway. In contrast, when autophagy is inhibited, intravesicular bodies are shunted toward the exosomal pathway, where they are secreted as TEXs (1,24,26).

## Autophagy Role in Immune Response

Tumor cells predominately express MHC-I; however, they can express MHC-II even when the tissue of origin does not (27). Elevated expression of MHC-II by both breast and ovarian tumors, which are typically associated with *BRCA1/2* mutation, has been correlated with better prognosis and overall survival advantage (28-30). Autophagy increases the tumor antigen supply of likely subclonal neoantigens for MHC-II presentation while decreasing expression of MHC-I by tumor cells (1,25,31). MHC-II expression of tumor neoantigens is a well-established immune therapeutic development direction (32). However, clonal neoantigen display is critical toward effective anticancer immune response (33). Overdisplay of subclonal neoantigens may dilute focused immune response to clonal neoantigens and even activate immune editing of clonal neoantigen effector cells (34,35). Despite autophagy’s role in refreshing the pool of available peptides to be presented by MHC-I (31), autophagy appears to recycle MHC-I more rapidly, resulting in decreased expression duration. Indeed, inhibition of autophagy related to gamma interferon expression augmented MHC-I expression in melanoma (36-38).

## Molecular Signaling and BRCA1/2 Function Related to Autophagy Regulation

Several molecular pathways overlap to control autophagy, particularly mTOR and Beclin1/ATG5. Type I PI3K/AKT/mTOR inhibits autophagy, whereas type III PI3K/vps34/Beclin1 activates autophagy (39). New evidence suggests that wild-type BRCA1 and BRCA2 proteins negatively regulate autophagy, with increased autophagy noted in mutant *BRCA1/2* gene samples as well as in *BRCA1/2* gene silencing studies (40-43). Coimmunoprecipitation studies of BRCA1 with Beclin1 show that when this complex is interrupted via a *BRCA1* mutation, the canonical pathway is enhanced because of the free action of Beclin1, and autophagy levels are increased (40). Additionally, in the role that BRCA1 functions as an antioxidant, the absence of functional BRCA1 increases reactive oxygen species, and autophagy increases in response to oxidative stress (41). Finally, BRCA1 interacts with PTEN, an inhibitor of the type I PI3K/AKT/mTOR pathway (44).

BRCA2 has been less thoroughly studied in relation to autophagy. Nonetheless, evidence supports a negative regulatory role of wild-type BRCA2 on autophagy (42,43). Knockdown of *BRCA2* via RNA interference in tumors with *BRCA1* allelic loss enhanced autophagy and mitophagy, a derivative of autophagy that recycles mitochondria (42). This same study suggested that PARP inhibitors, particularly olaparib whose mechanism depends on interrupted BRCA1/2 function, have enhanced function in the context of increased autophagy (42). It remains unknown if autophagy is similarly upregulated regardless of *BRCA* gene variant or in tumors that are homologous recombination deficient. It is known that autophagy is required for homologous recombination to take place, and in the absence of autophagy, DNA damage accumulates, resulting in cell death (45).

## *BRCA1/2* Mutation Role in Autophagy and Cancer Immunity

Autophagy and exosome transport are multifaceted processes, and their role in cancer development, progression, and immunity relates to *BRCA1/2* expression and genetic stability (31). BRCA1/2 wild-type expression decreases autophagy, whereas *BRCA1/2* mutation enhances autophagy activity (1,40). Tumors with increased autophagy due to defective BRCA1/2 demonstrate increased MHC-II expression of tumor antigens (1,40,42). However, tumors with *BRCA1/2* mutation also exhibit increased estimated glomerular filtration rate (EGFR) expression, which decreases MHC expression compared with wild-type tumors (46,47). EGFR also directly regulates autophagy in a context-dependent manner, which may be depend on localization (48). Through association with Beclin1, inactive EGFR can decrease autophagy; however, when localized to the endosome, EGFR can also initiate autophagy (49,50). The complex correlation between autophagy, EGFR, and MHC expression warrants further research.

It is hypothetically possible that germline vs somatic *BRCA* mutation status may affect immune response differently. In patients with germline mutations, it is presumed that immune and cancer cells would be impacted by altered BRCA functioning, potentially resulting in increased autophagy across the board. It has been shown that *BRCA* heterozygous mice have decreased white blood cells and lymphocytes. The same study also showed that heterozygous *BRCA1* carriers are more at increased risk for chemotherapy-associated hematopoietic complications (51). Although this could be due to increased DNA damage in these cells, it may be linked to autophagy. Further investigation would be needed to validate this hypothesis. Of interest, immune cells with increased autophagy express both increased MHC-I and MHC-II (1,31,52,53). This is explained by increased dendritic cell participation in cross-presentation (1,25,54). Complementary to autophagy, cross-presentation allows extracellular material to be presented by MHC-I (autophagy places intracellular material on MHC-II) (1,25). Moreover, cross-presentation allows DCs to accept tumor antigens from TEXs and to present them on MHC-I and II on the DC surface (1,25). Because DCs express costimulatory molecules, cross-presentation allows antigens from TEXs to elicit a more effective immune response than the same antigen could in the absence of DCs (1,54). In contrast, we presume that patients with somatic *BRCA1/2* mutations demonstrate the effect of autophagy induction exclusively in the tumor cells containing the *BRCA1/2* mutation.

BRCA1/2 activity on autophagy, however, is complicated. For example, increased autophagy in *BRCA1/2* mutant or dysfunctional tumor cells destabilizes the immunological synapse between cytolytic immune cells and their targets, consequently disrupting transfer of cytotoxic molecules (55). In particular, autophagy increases degradation of granzyme B and connexin-43, which are 2 important molecules in the tumor-killing pathway enacted by cytotoxic T lymphocytes (CTLs) and natural killer (NK) cells (55-59). Moreover, inhibition of autophagy restores cytolytic activity in cell populations that are resistant to CTL- and NK-mediated killing (57,60,61). Conversely, upregulation of autophagy decreases CTL- and NK-mediated killing, particularly during the endothelial-mesenchymal transition, which is vital for metastasis (62).

Autophagy also suppresses the immune response indirectly via its effects on the exosomal pathway. Presumably, expressive *BRCA* wild-type tumor cells have increased exosome secretion as opposed to *BRCA*-mutation tumor cells. With low levels of autophagy, homeostasis shifts toward the exosomal pathway, with subsequently increased release of TEXs (24,26). Although TEXs represent a vehicle for transporting tumor antigens to distant antigen presenting cells (APCs), they also have a major role in immunosuppression of the anticancer immune response (54). This is accomplished through expression of immunosuppressive proteins including TGF-β, IL-6, and PGE2 (63-66). Furthermore, TEXs frequently carry miRNAs that suppress expression of immunostimulatory genes in target cells (54,67,68). Other immunosuppressive effects include redirection of myeloid precursors toward myeloid-derived suppressor cells, along with inhibited differentiation of DCs from precursors (63,69). Along these same lines, TEXs prevent the maturation of immature DCs, alter pattern recognition receptors to impair antigen recognition, and reduce DC production of immunostimulatory cytokines including TNF-α and IL-12 (66,70). Furthermore, TEXs induce the CD14+ HLA-DR^-/low^ monocyte subtype, which suppresses T-cell proliferation and cytotoxicity (64). Beyond monocytes, TEXs negatively impact the development of NK cells and CTLs (71-76). Finally, TEXs induce the regulatory subtypes of T and B cells (77-80). In sum, TEXs have a complicated impact on anticancer immunity, and they are increased in settings with low autophagy. This includes wild-type expression of *BRCA1/2* (40-43).

## Clinical Relevance

Tumor expression of PD-L1, an immune checkpoint, is inversely related to autophagy (31). With activation of autophagy, PD-L1 levels are decreased. In contrast, inhibition of autophagy results in increased expression of PD-L1 (31). Similarly, approximately 90% of human lung cancer samples with increased PD-L1 expression showed inhibition of autophagy, whereas 83% of tumors with negative PD-L1 had increased autophagy (81). Likewise, blocking PD-L1 disinhibited autophagy, whereas increasing PD-L1 signaling inhibited autophagy (82). Thus, it is expected that *BRCA* wild-type patients would have increased PD-L1 signaling, whereas *BRCA* mutant would have decreased PD-L1 signaling. Therefore, *BRCA* mutation status may relate to clinical benefit of immune checkpoint inhibitor (ICI) therapy.

In the *BRCA1/2* wild-type population, for example, patients would likely derive benefit from therapies that introduce tumor antigens to the immune system via an alternative mechanism, for example, use of Vigil, a personalized neoantigen-educating immunotherapy as opposed to checkpoint-inhibitor therapy, which does not modulate neoantigen expression and would have limited activity in lower clonal neoantigen expressive tumors (83-86). Vigil is an experimental therapeutic that educates the immune system via an autologous tumor vaccine along with plasmid DNA-encoding upregulators of the immune system (granulocyte-macrophage colony-stimulating factor) and blocking downregulators of the immune system (furin, which activates TGF-β, a major immunomodulatory molecule) (87). In clinical trials involving melanoma, Ewing sarcoma, ovarian cancer, and other solid tumors, Vigil was well tolerated (83,87-91). In solid tumors, improved clinical outcomes related to Vigil treatment were correlated with γ-IFN-ELISPOT positive response, indicating that Vigil is able to activate a durable immune response. Combination of Vigil followed by immune checkpoint inhibitor may sensitize patients to checkpoint inhibitors by priming T cells to the relevant clonal tumor neoantigens.

## Conclusion

Increased tumor cell autophagy (as in *BRCA* mutant) likely leads to enhanced presentation of subclonal neoantigens to the immune system but impaired cytotoxic killing. Conversely, inhibited tumor autophagy (as in *BRCA* wild-type) likely causes lower tumor antigen presentation but may preserve clonal neoantigen display supporting increased target-directed susceptibility to cell-mediated destruction. Consequently, *BRCA* status may impact a patient’s response to certain immunotherapies. Going forward, research involving clinical therapeutic measures that involve and/or modulate BRCA1/2 signaling as involved in autophagy control and relationship to antigen presentation is justified.

## Funding

The authors have no funding to disclose.

## Notes

**Role of the funder:** Not applicable.

**Author contributions:** Susan Morand: Contributed to the research and writing of the manuscript. Laura Stanbery: Contributed to the research and writing of the manuscript. Adam Walter: Contributed to the writing and editing of the manuscript. Rodney Rocconi: Contributed to the writing and editing of the manuscript. John Nemunaitis: Initiated review design, contributed to article design, writing, and editing.

**Disclosure:** The authors have no conflicts of interest to disclose.

**Acknowledgments:** The authors would like to acknowledge Brenda Marr for her competent and knowledgeable assistance in the preparation of the manuscript.

**Prior presentations**: None

## Data availability

 Not applicable.
